# MicroRNAs in Muscle: Characterizing the Powerlifter Phenotype

**DOI:** 10.3389/fphys.2017.00383

**Published:** 2017-06-07

**Authors:** Randall F. D'Souza, Thomas Bjørnsen, Nina Zeng, Kirsten M. M. Aasen, Truls Raastad, David Cameron-Smith, Cameron J. Mitchell

**Affiliations:** ^1^Liggins Institute, University of AucklandAuckland, New Zealand; ^2^Department of Public Health, Sport and Nutrition, Faculty of Health and Sport Sciences, University of AgderKristiansand, Norway; ^3^Department of Physical Performance, Norwegian School of Sport SciencesOslo, Norway

**Keywords:** microRNA, resistance training, gene expression, skeletal muscle, mRNA

## Abstract

Powerlifters are the epitome of muscular adaptation and are able to generate extreme forces. The molecular mechanisms underpinning the significant capacity for force generation and hypertrophy are not fully elucidated. MicroRNAs (miRs) are short non-coding RNA sequences that control gene expression via promotion of transcript breakdown and/or translational inhibition. Differences in basal miR expression may partially account for phenotypic differences in muscle mass and function between powerlifters and untrained age-matched controls. Muscle biopsies were obtained from m. vastus lateralis of 15 national level powerlifters (25.1 ± 5.8 years) and 13 untrained controls (24.1 ± 2.0 years). The powerlifters were stronger than the controls (isokinetic knee extension at 60°/s: 307.8 ± 51.6 Nm vs. 211.9 ± 41.9 Nm, respectively *P* < 0.001), and also had larger muscle fibers (type I CSA 9,122 ± 1,238 vs. 4,511 ± 798 μm^2^ *p* < 0.001 and type II CSA 11,100 ± 1,656 vs. 5,468 ± 1,477 μm^2^ *p* < 0.001). Of the 17 miRs species analyzed, 12 were differently expressed (*p* < 0.05) between groups with 7 being more abundant in powerlifters and five having lower expression. Established transcriptionally regulated miR downstream gene targets involved in muscle mass regulation, including myostatin and MyoD, were also differentially expressed between groups. Correlation analysis demonstrates the abundance of eight miRs was correlated to phenotype including peak strength, fiber size, satellite cell abundance, and fiber type regardless of grouping. The unique miR expression profiles between groups allow for categorization of individuals as either powerlifter or healthy controls based on a five miR signature (miR-126, -23b, -16, -23a, -15a) with considerable accuracy (100%). Thus, this unique miR expression may be important to the characterization of the powerlifter phenotype.

## Introduction

Powerlifting is a competitive sport which requires maximal lifts for three multi-joint exercises. Powerlifters display an extreme capacity to generate muscle force relative to their body size. Self-selection and genetic predisposition undoubtedly play a part in becoming an elite powerlifter. However, extensive resistance training is required to make a competitive powerlifter (Ostrander et al., 2009; Pitsiladis et al., 2013). Regardless of the relative importance of genetics and training, powerlifters embody a unique phenotype, which can aid in understanding the molecular mechanisms which regulate muscle mass and strength.

Powerlifters are stronger relative to body size in comparison to untrained individuals. Theoretically, this greater strength is due to a combination of increased fiber area, altered muscular architecture and an improved ability to activate the muscle (Kawakami et al., 1993; Brechue and Abe, 2002; Matta et al., 2011). However, the relative importance of each of these factors is not fully elucidated (Edman, 1979; Narici et al., 1989).

Muscle hypertrophy occurs through a net accretion of muscle contractile proteins. Chronic resistance training promotes muscle anabolism via a complex interaction of multiple competing pathways (Marcotte et al., 2015). A number of myogenic regulatory factors including MyoD and MyoG are upregulated in individuals after chronic resistance exercise (Kosek et al., 2006) and are involved in myogenic remodeling and programming following exercise (Yablonka-Reuveni et al., 1999). Conversely, resistance exercise reduces expression of some genes which promote muscle degradation such as myostatin and atrogin1, while increasing the expression others such as MuRF1 (Zanchi et al., 2009; Fernandez-Gonzalo et al., 2013). Satellite cells, which are PAX7+ multipotent cells resident in the stem cell niche between mature muscle fibers and the basal lamina (Yin et al., 2013), are involved in the maintenance and repair of damaged fibers, while also being capable of fusing, to increase fiber number or supporting hypertrophy via nuclear addition (Petrella et al., 2008; McCarthy et al., 2011; Roberts et al., 2015). PAX7 mRNA and positive cells expression have been shown to increase with both acute and chronic resistance exercise (Nielsen et al., 2012; Bellamy et al., 2014; Nederveen et al., 2017).

Central in the coordinated regulation of gene expression are microRNAs (miRs), small non-coding RNAs, that selectively bind, inhibiting translation or promote degradation of targeted mRNAs (Ambros et al., 2003; Mathonnet et al., 2007; Townley-Tilson et al., 2010). Hundreds of different miRs are expressed in human tissue, with many found to be highly enriched in specific tissue types (Landgraf et al., 2007; Walden et al., 2009). To date only a few studies have examined the effect of anabolic stimuli such as resistance exercise or feeding on muscle miR profiles (Drummond et al., 2008; Davidsen et al., 2011; Tonevitsky et al., 2013; Zacharewicz et al., 2013). Of these analyses, 8 miRs (miR-1, -133a, -133b, -206, -208a, -208b, -486, and -499a) have been consistently identified as myogenic miRNAs (myomiRs) that are vastly more abundant within skeletal muscle compared to other tissues (McCarthy and Esser, 2007; van Rooij et al., 2009; Small et al., 2010). These myomiRs have been implicated in a variety of roles within muscle. For example, miR-1, -133a, and -206, are all involved in the regulation of Pax7 and are themselves regulated by downstream genes such as MyoD and MyoG (Chen et al., 2006; Ikeda et al., 2009; Braun and Gautel, 2011). miR-208a, -208b, and 499a inhibit myostatin and thus are important in muscle catabolism (Drummond et al., 2009; Hitachi and Tsuchida, 2014). In addition, miR-499a inhibits Sox-6 expression which plays a role in the conversion between muscle fiber types (McCarthy, 2011).

Further to these established myomiRs, there is evidence from either myogenic cell lines, animal and human studies, of other miRs that may exert crucial roles in control of muscle hypertrophy, atrophy, myogenesis, and apoptosis via cell cycle regulation (Muscat and Dressel, 2000; Naguibneva et al., 2006; Fish et al., 2008; Wang et al., 2008; Williams et al., 2009; Aqeilan et al., 2010; Nakasa et al., 2010; Nan et al., 2010; Dey et al., 2011; Sun et al., 2012). From this increasing literature, only those miRNAs that have been demonstrated from both mechanistic analysis in cell lines and *in-vivo* were selected as being key candidates for analysis. This included an additional subset of 9 miRs [miR-30b (Naguibneva et al., 2006; Nielsen et al., 2010), -148b (Li and Xi, 2011; Gastebois et al., 2016), -145 (Cordes et al., 2009; Sachdeva et al., 2009), -23a (McCarthy, 2014b; Kapchinsky et al., 2015), -23b (Wang, 2013; Režen et al., 2014), -126 (Fish et al., 2008; Wang et al., 2008), -15a, -16 (Bandi and Vassella, 2011; Musumeci et al., 2011; Sun et al., 2012; Yin et al., 2012), and -451a (Mercken et al., 2013; Zang et al., 2015)].

Powerlifters are capable of generating very high relative muscular forces due to greater muscle size and quality than the general population (MacDougall et al., 1982). It is unknown if the basal expression of miRs differs between powerlifters and healthy controls. Further, it is unclear if miR expression may partially regulate the expression of genes related to the regulation of muscle mass. Hence, the aim of this study was to quantitatively analyse using real-time PCR, the regulation of the extended list of key myomiRs and key myogenic regulatory mRNA species in both elite powerlifters and healthy age matched controls. It was hypothesized that muscle specific miRs with defined roles in muscle mass regulation are differentially expressed between these distinctive muscular phenotypes, and that these differences will relate to the expression of downstream mRNAs.

## Methods

### Participants

Thirteen recreationally active young students 24.3 ± 1.8 years and 15 elite Norwegian Powerlifters aged 23.5 ± 3.1 years were recruited (Table 1). Exclusion criteria were any injuries of the musculo-skeletal system that could prevent the participant from exerting maximal force, use of medication and the use of anabolic steroids. Subjects were asked to refrain from any strenuous exercise for 72 h prior to the study day. The study was complied with the standards set by the Declaration of Helsinki and was reviewed by the Regional Committee for Medical and Health Research Ethics (REC South-East). The nature and goals of the study were thoroughly explained, and all subjects provided a written informed consent.

**Table 1:** Participant Characteristics.

	**Healthy controls**	**Power lifters**
Age (years)	24.3 ± 1.8	23.5 ± 3.1
Height (m)	178.6 ± 7.4	176.4 ± 7.1
Weight (kg)	77.4 ± 12.1	94.8 ± 16.7
BMI (kg/m^2^)	24.1 ± 2.3	30.4 ± 4.3

*Values presented as means ± SD, n = 13 controls and n = 15 power lifters*.

### Muscle strength

Isokinetic torque of the knee extensor was measured at 60° per second over a range of 70° (from 20° to 90° when 0° is fully extended) using a dynamometer (HUMAC 2009NOMR CSMi. Testing and Rehabilitation System, USA). Participants were strapped to the dynamometer chair with two belts crossing over their chest. Hands were placed on these belts to ensure isolation of knee extensor muscles. Participants repeated the test three times after four warm up attempts with strong verbal motivation from the same individual with the best value being recorded. To correct for variance between body size between groups, peak torque was normalized to height as a form of allometric scaling of strength as proposed by Jaric (2003).

### Muscle biopsy sampling

Muscle biopsies (200–300 mg) were obtained from m. vastus lateralis using a 6 mm sterile Bergström needle under local anesthesia (Xylocain-adrenaline, 10 mg/ml +5 mcg/mL, AstraZeneca, Södertälje, Sweden). Connective tissue and fat were dissected away before a bundle of fibers identified for later immunohistochemical analyses was mounted in OCT Embedding Matrix (Tissue-tek, O.C.T. compound, Sakura, USA) and immediately frozen in isopentane, which was pre-cooled (~−140°C) with liquid nitrogen and stored at −80°C for later analysis. A ~20 mg piece was snap frozen in liquid nitrogen for RNA analysis.

### miR/mRNA isolation

Total RNA was extracted from ~20 mg of muscle tissue using the AllPrep® DNA/RNA/miRNA Universal Kit (QIAGEN GmbH, Hilden, Germany) following the manufacturer's instructions as described by Figueiredo et al. (2016).

### miR cDNA/RT-PCR

10 ng of input RNA was used for cDNA synthesis using TaqMan™ Advanced miRNA cDNA Synthesis Kit (Thermo Fisher Scientific, Carlsbad, CA, USA), miR abundance were measured by RT-PCR on a QuantStudio 6 (Thermo Fisher Scientific, Carlsbad, CA, USA) using Applied Biosystems Fast Advanced Master Mix (Thermo Fisher Scientific, Carlsbad, CA, USA).

Target miRNAs were miR-15a-5p, -23a-5p, -23b-5p, -499a-3p, -206, -208a-3p, 208b-3p, -451a, -486-5p, -126-3p, -1-3p, -133a-3p, -133b, -148b-3p, -30b-3p, -145-5p, and -16-5p Thermo Fisher Scientific, Cat# A25576, Carlsbad, CA, USA) (Supplementary Table 1). The geometric mean of three reference miRNAs (miR-361-5p, -320a, -186-5p; Vandesompele et al., 2002) was used for normalization based on miRNAs that showed the least variation amongst the participants between and within groups. The abundance of miRs were measured using the 2^(−ΔCT)^ method (Schmittgen and Livak, 2008).

### mRNA cDNA /RT-PCR

1500 ng of input RNA was used for cDNA synthesis using the High–Capacity RNA-to-cDNA™ kit (Life Technologies, Carlsbad, CA). Messenger RNA (mRNA) was measured by RT-PCR on a LightCycler 480 II (Roche Applied Science, Penzberg, Germany) using SYBR Green I Master Mix (Roche Applied Science). Target mRNAs were decided based on previously published pubmed literature that associated targeted miRs with muscle regulatory genes. These included, cMYC, Myogenic Differentiation 1 (MyoD), Forkhead Box O3 (FOXO3), PAX7, Cyclin D1 (CCND1), Myogenin (MyoG), Neural Cell Adhesion Molecule (NCAM), Atrogin-1, Muscle-Specific RING Finger Protein 1 (MuRF1), Vascular Endothelial Growth Factor (VEGF), Myostatin, Histone Deacetylase 4 (HDAC4), Bone Morphogenetic Protein 2 (BMP2), SRY (Sex Determining Region Y)-Box 6 (SOX6), Phosphatase and tensin homolog (PTEN), Serum response factor (SRF), SPRED1, PAX3, and Forkhead Box 01 (FOXO1). Primers were designed using BLAST software (sequences in Supplementary Table 2). The geometric mean of four reference genes was used for normalization (Vandesompele et al., 2002). The recently proposed human reference genes (Eisenberg and Levanon, 2013), endoplasmic reticulum membrane protein complex subunit 7 (*EMC7*), valosin-containing protein (VCP), charged multivesicular body protein 2A (*CHMP2A*), and chromosome 1 open reading frame 43 (*C1orf43*) were identified as the least variable and therefore, used as reference genes (Supplementary Table 3). Standard and melting curves were performed for every target to confirm primer efficiency and single-product amplification. The abundance of mRNAs were measured using the 2^(−ΔCT)^ method (Schmittgen and Livak, 2008).

### Immunohistochemical staining

Muscle biopsies were cut to 8 μm thick cross sections at −20°C using a cryostat (CM 1950, Leica Biosystems GmbH, Nussloch, Germany) and mounted on microscope slides (Superfrost Plus, Menzel-Glaser, Brouschweig, Germany). Glass slides from the freezer were air dried at room temperature for 10 min and a PAP-pen (OmmEdge PEN. Vevtor Laboratories, Inc) was used to draw a lipid ring around sections. Next, primary antibodies and stains were applied for 45 min incubation in 1% Bovine Serum Albumin (BSA) (Dako, 10082504) and PBS-t solution (QC213624, Thermo Fisher Scientific, Carlsbad, CA, USA). BSA was removed and the slide dried using lint-free paper towel (Kimtect Science, Precision Wipes Tissue Wipers) to prepare for secondary antibody application. Specific secondary antibodies [Alexa-488 goat anti-mouse (Biotium, Inc, Hayward, CA, USA, 1:200) and CF-594 goat anti-rabbit (Biotium, 1:200) were applied after each primary antibody. Sections were mounted with a fluorescent anti-fade containing DAPI (for nuclear staining) (Invitrogen, 1266174, Life Technologies, Denmark, Naerum, Denmark) and coverslip were pasted together with slides and stored protected from light in a fridge (5°C). Satellite cells were visualized with an antibody against PAX7 (DSHB, 1:100) and laminin (Dako, 20025756, 1:400) as well as DAPI-stains (for nuclear staining) (Invitrogen, 1266174, Life Technologies, Denmark, Naerum, Denmark), while serial sections for MCH-II (DSHB, 1:1,000) and dystrophin (ABCam, ab15277), 1:500] and were added onto a separate slide for distinction of the myofiber border and myofibers type II.

Stained biopsies were visualized on a computer screen using a light microscope (Olympus BX61, Japan) connected to a fluorescent light (EXFO, Xl120PC-Q, Canada) and was used to quantify the sections. The microscope was also connected to a digital camera (Olympus DP72, Japan) that took pictures at 20x zoom of the sections. All morphometric analysis was performed in Cell-F (Olympus, Japan), TEMA (ChekVision, Hadsund, Denmark) and ImageJ (version 2.0.0-rc-41/1.5 d, National Institutes of Health, Bethseda, MD, USA). Type I (unstained) and type II (stained) myofibers were differentiated, and myofiber area was determined. On average 543 ± 241 myofibers were analyzed per biopsy sample for the assessment of muscle fiber area. Satellite cells and myonuclei were identified using the following criteria: SC had to stain positive for PAX7 and be placed within the basal lamina; nuclei with a subordinate placement were considered myonuclei. The number of PAX7 positive satellite cells and myonuclei are presented relative to the number of type I and II myofibers. Myonuclear domain is expressed as the area of each fiber type supported by a single myonucleus. A total of 50 myofibers for each fiber type were analyzed for quantification of myonuclei, in accordance with previous methods (Mackey et al., 2009). The same investigator performed all analyses manually.

### Statistical analysis

Statistical analysis was performed using Graph Pad Prism Software (GraphPad Software Inc., La Jolla, CA). Differences between controls and powerlifters were determined using Student's *t*-test. Multiple comparison corrections were undertaken using false discovery rates where *p* < *q* was determined as significant with alpha set at *p* < 0.05 (Supplementary Table 4). Linear regression was performed using measures of phenotype including peak strength, fiber CSA, fiber area per satellite cell and fiber type as dependant variables with miRs as independent variables. Only miRs which independently correlated with the dependant variable and did not correlate with expression of any miRs already in the model were included in the linear regression models. Step wise discriminant analyses was performed using IBM SPSS for Windows Version 23 (IBM Corp. USA) to determine the ability of miRs to distinguish between powerlifters and controls, as per (Margolis et al., 2016). Receiver operating characteristic curves (Prism software) were then used to determine the area under the curve for each of the potential of miR-126, -23a, -16, -23a, and -15a in order to correctly identify the powerlifter phenotype. Prism software was also used to generate graphs. Data are shown as means ± *SD*. Statistical significance was accepted at *p* < 0.05.

## Results

### Skeletal muscle strength and histology

**Figure 1:**
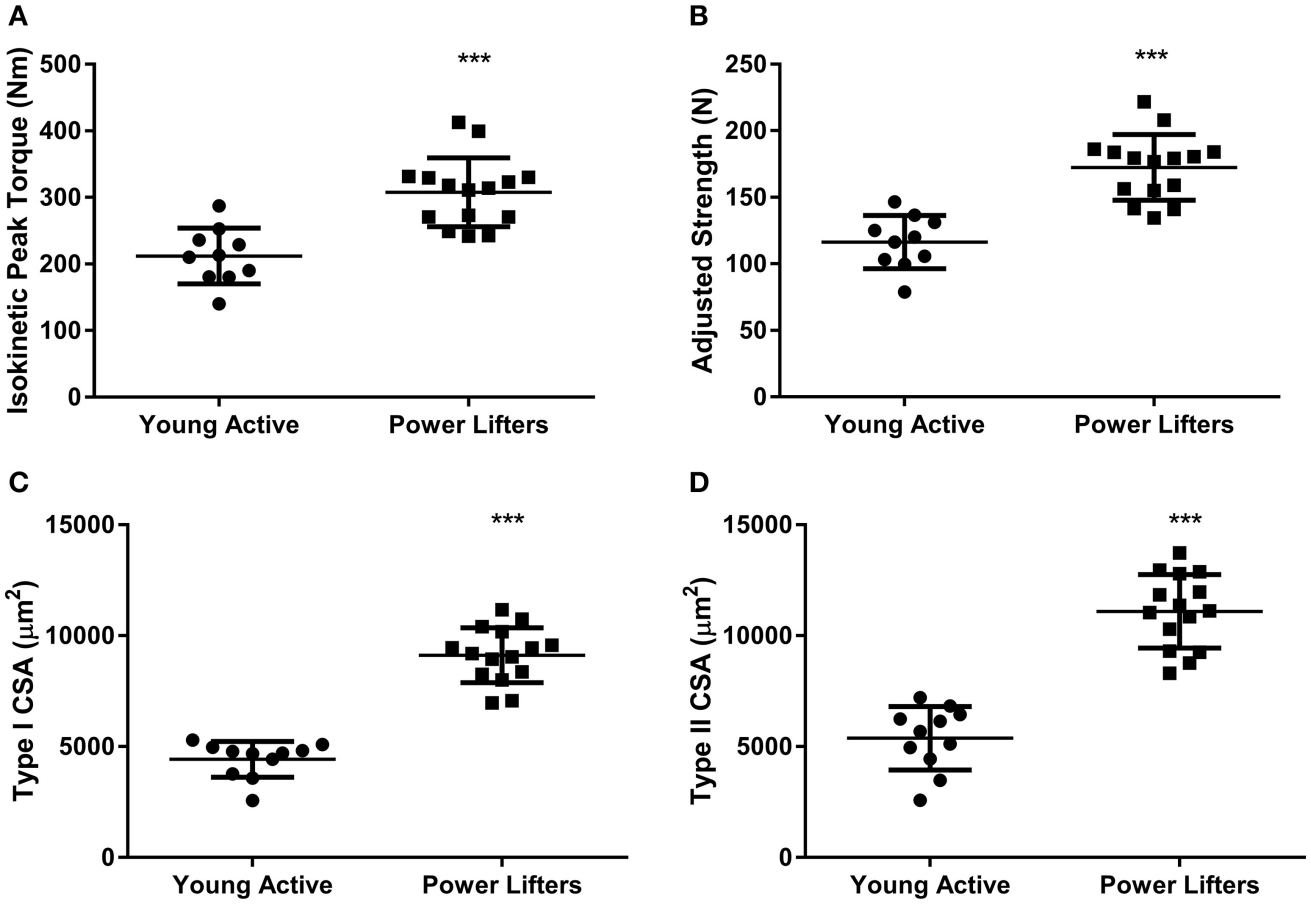
Phenotype. **(A)** Peak knee extension torque (Nm). **(B)** Peak knee extension torque corrected for body size **(C)** Type I CSA (μm^2^). **(D)** Type II CSA (μm^2^). (^*^difference between power lifter and controls *p* < 0.05, ^**^difference between power lifter and controls *p* < 0.005, and ^***^ difference between power lifter and controls *p* < 0.001). Data expressed are expressed as means ± *SD*.

**Figure 2:**
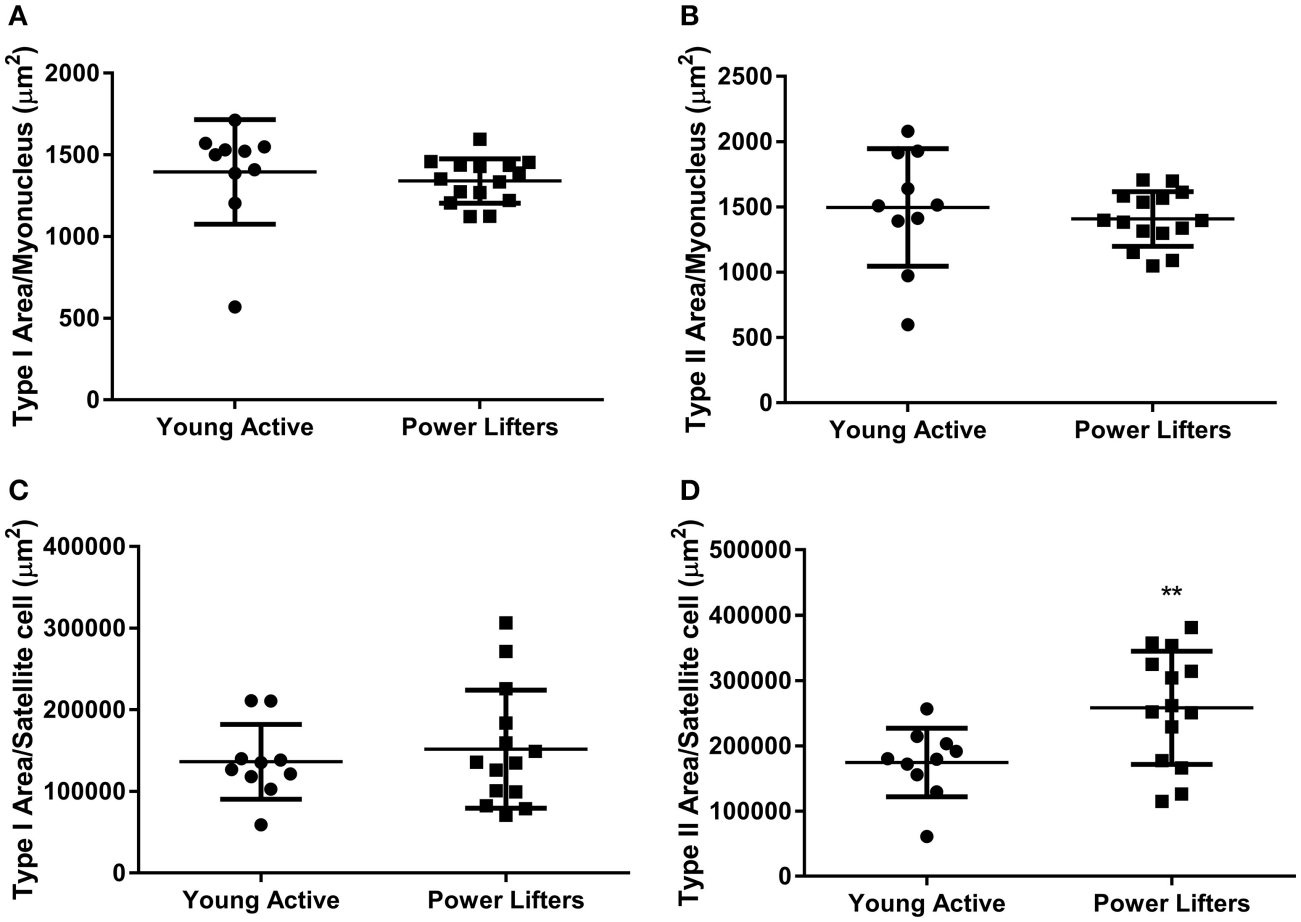
Immunohistochemistry. Area of fiber per nucleus. **(A)** Type I (μm^2^). **(B)** Type II (μm^2^). Area of fiber per satellite cell. **(C)** Type I (μm^2^). **(D)** Type II (μm^2^). (^*^difference between power lifter and controls *p* < 0.05, ^**^difference between power lifter and controls *p* < 0.005, and ^***^difference between power lifter and controls *p* < 0.001). Data expressed are expressed as means ± *SD*.

The powerlifters were stronger (*p* < 0.001) than the healthy control group. This difference was maintained when corrected for body size (*p* < 0.001; Figures 1A,B). Powerlifters also had larger muscle fibers (*p* < 0.001) of both fiber types (Figures 1C,D). The area per myonucleus (myonuclear domain) was not different between groups irrespective of fiber type (type I *p* = 0.555 and type II *p* = 0.515; Figures 2A,B). No difference between area of type I fibers per satellite cell was seen between groups (*p* = 0.560) however, the powerlifters had increased type II area per satellite cell (*p* = 0.007; Figures 2C,D; Table 2).

**Table 2:** Myonuclei and satellite cells.

	**Healthy controls**	**Power lifters**	***p*-values**
Fiber ratio (Type I : II)	1.1 ± 0.4	0.8 ± 0.3	0.022^*^
Myonuclei/Type I Fiber	3.3 ± 0.4	6.8 ± 1.3	<0.001^*^
Myonuclei/Type II Fiber	3.6 ± 0.4	7.8 ± 0.9	<0.001^*^
Satellite cell/Type I Fiber	0.036 ± 0.007	0.071 ± 0.033	0.003^*^
Satellite cell/Type II Fiber	0.036 ± 0.014	0.049 ± 0.021	0.111

*Values presented as means ± SD, n = 13 controls and n = 15 power lifters*.

* *difference power lifters and controls p < 0.05*.

### miR and gene expression

**Figure 3:**
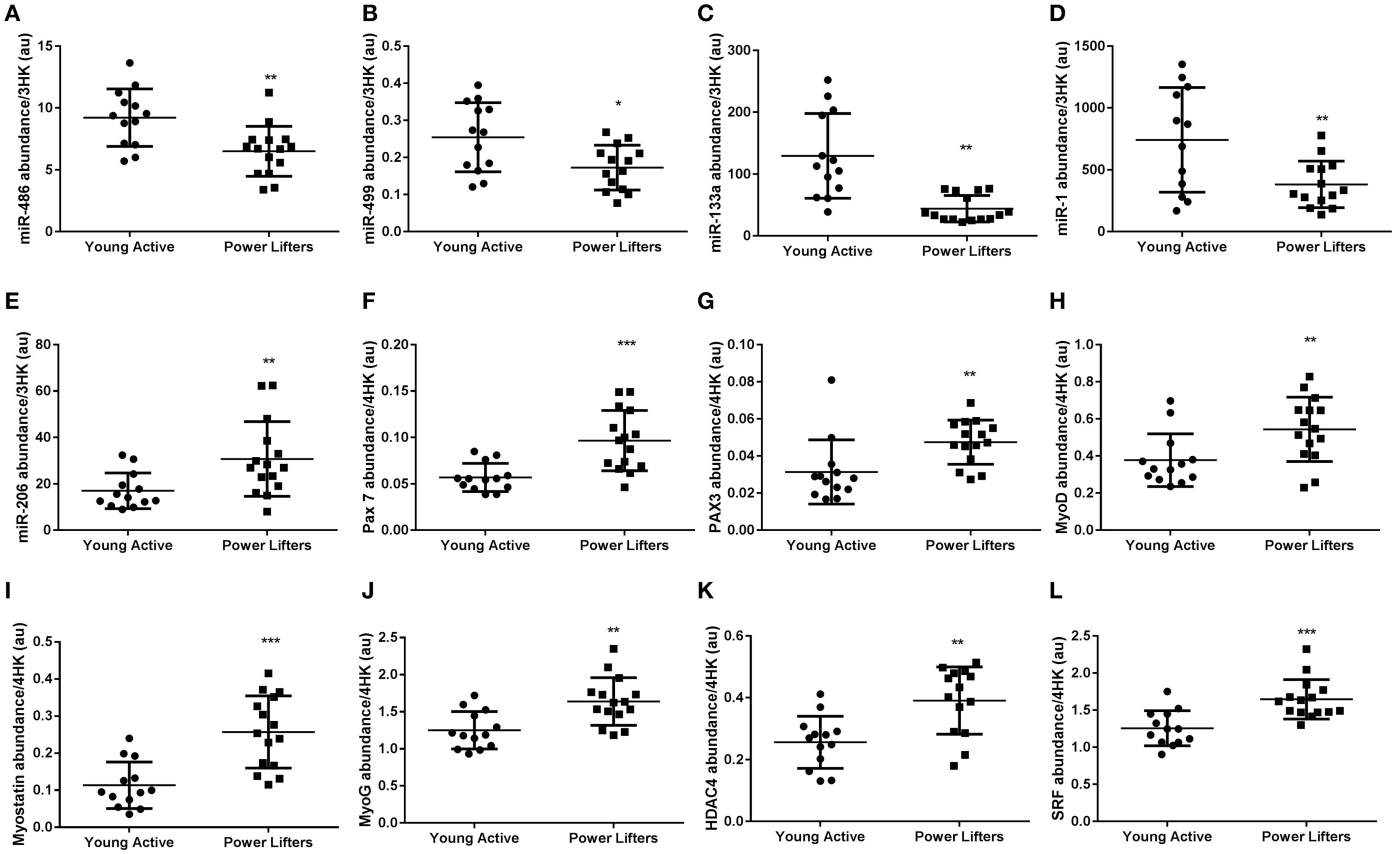
MyomiR and related gene abundance. **(A)** miR-486, **(B)** miR-499a, **(C)** miR-133a, **(D)** miR-1, **(E)** miR-206, **(F)** PAX-7 mRNA, **(G)** PAX3 mRNA, **(H)** MyoD mRNA, **(I)** Myostatin mRNA, **(J)** MyoG mRNA, **(K)** HDAC4 mRNA, **(L)** SRF mRNA. miRs normalized to geomean of 3 endogenous stable miRs, mRNAs normalized to geomean of 4 housekeepers. (^*^difference between power lifter and controls *p* < 0.05, ^**^difference between power lifter and controls *p* < 0.005, and ^***^difference between power lifter and controls *p* < 0.001). Data expressed are expressed as means ± *SD*.

Five of the seven myomiRs measured were differentially abundant between groups. Four were elevated (miR-486 *p* = 0.003, -499a *p* = 0.012, -133a *p* < 0.001 and -1 *p* = 0.008), one lower (miR-206 *p* = 0.009) in the healthy controls compared to the powerlifters, two were similarly expressed (miR-208a *p* = 0.71 and -208b *p* = 0.496) between groups. MyomiRs have putative roles in the control of known muscle mass regulators such as PAX7 (*p* < 0.001), PAX3 (*p* = 0.008), MyoD (*p* = 0.011), Myostatin (*p* < 0.001), MyoG (*p* = 0.002), HDAC4 (*p* = 0.001), SRF (*p* < 0.001), and SOX6 (*p* = 0.008) which were all more abundant in the powerlifters while PTEN (*p* = 0.302) transcript abundance was not different between groups (Figure 3).

**Figure 4:**
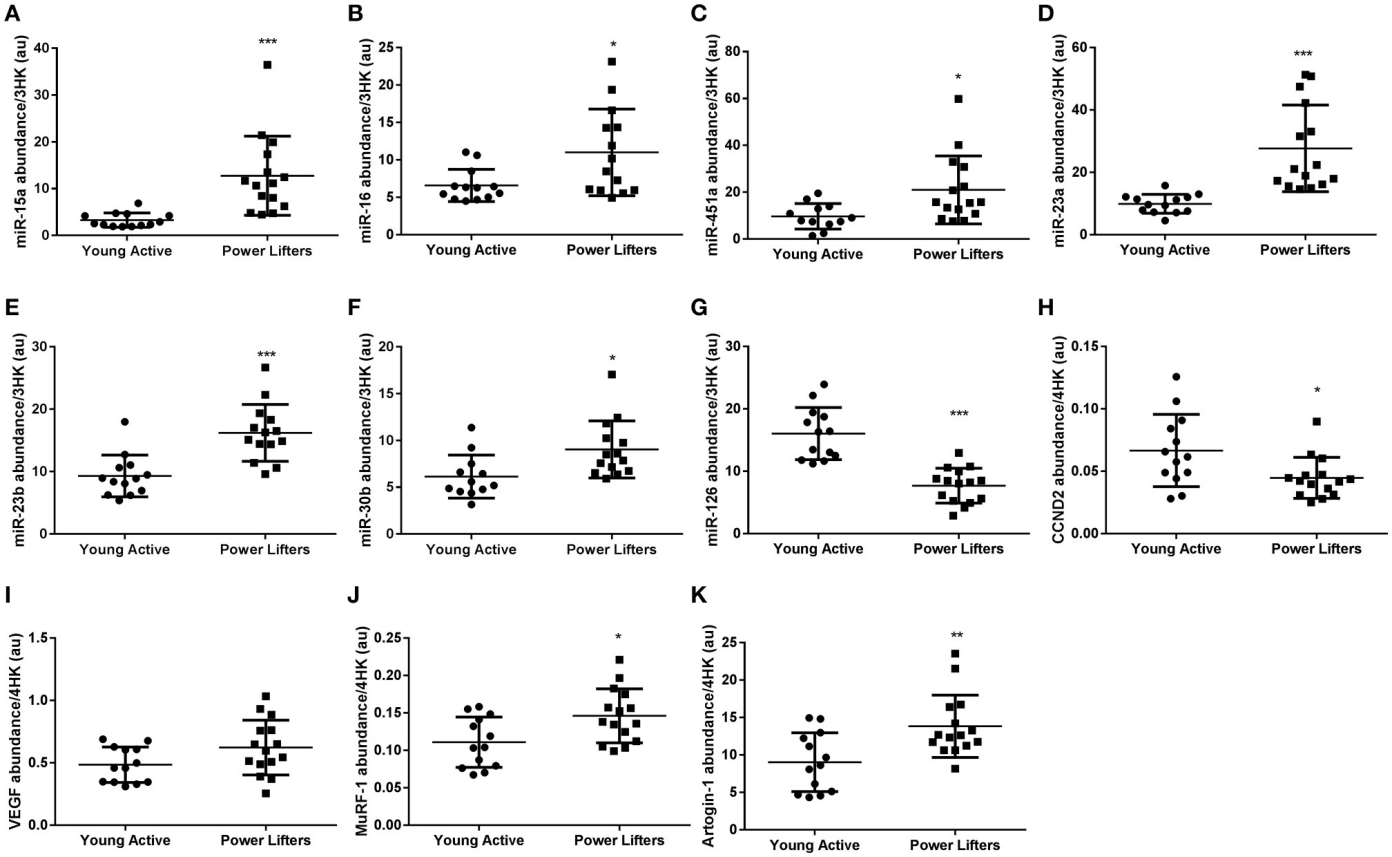
Other muscle miRs and related gene abundance. **(A)** miR-15a, **(B)** miR-16, **(C)** miR-451a, **(D)** miR-23a, **(E)** miR-23b, **(F)** miR-30b, **(G)** miR-126, **(H)** CCND2 mRNA, **(I)** VEGF mRNA, **(J)** MuRF-1 mRNA, **(K)** Atrogin-1 mRNA. miRs normalized to geomean of 3 endogenous stable miRs, mRNAs normalized to geomean of 4 housekeepers. (^*^difference between power lifter and controls *p* < 0.05, ^**^difference between power lifter and controls *p* < 0.005, and ^***^difference between power lifter and controls *p* < 0.001). Data expressed are expressed as means ± *SD*.

miR-15a (*p* < 0.001), -16 (*p* = 0.016), and -451a (*p* = 0.017) were elevated in the powerlifters compared to the healthy controls (Figures 4A–C). Downstream target cyclin D1 (*p* = 0.198) was not different between groups while D2 (*p* = 0.019) (Figure 4H) was less abundant in the powerlifter group. Futhermore, miR-15a and -16 have reported roles in inhibiting angiogenesis via action on VEGF which trended toward being more abundant in powerlifters (*p* = 0.064; Figure 4I). miR-145 showed no difference between groups (*p* = 0.730).

miR-23a (*p* < 0.001) and -23b (*P* < 0.001) are both inhibitors of catabolic gene expression (Wada et al., 2011) and were found to be elevated in the powerlifter group (Figures 4D,E). Catabolic genes FOXO1/3 (*p* = 0.244, *p* = 0.213) were unchanged between groups while MuRF1 (*p* = 0.013) and Atrogin1 (*p* = 0.004) all showed increased expression in powerlifters compared to controls (Figures 4J,K). The upstream regulator c-MYC was expressed similarly between groups (*p* = 0.693).

miR-30b expression (*p* = 0.013) was higher in powerlifters compared to controls (Figure 4F). miR-126 (*p* < 0.001) had lower expression in powerlifters compared to controls (Figure 4G). SPRED-1 (*p* = 0.083), an inhibitory target of miR-126 upstream of VEGF showed no difference between groups.

### Correlation analysis

miRNAs were correlated against phenotype with both groups combined. With multiple regression analyses, we see a range of significant in relationships as described in Table 3.

**Table 3:** Phenotype vs. miR expression correlations.

	***R*^2^**	**β**	***P*-value**
Strength (miR-133a)	0.368	−0.606	0.001
Corrected strength	0.47	–	0.001
miR-133a	–	−0.568	0.001
miR-486	–	−0.273	0.043
Type I CSA	0.63	–	<0.001
miR-206	–	0.446	0.002
miR-16	–	0.269	<0.001
miR-133a	–	−0.398	0.011
Type II CSA	0.668	–	<0.001
miR-206	–	0.351	0.007
miR-133a	–	0.446	0.018
miR-486	–	−0.277	<0.001
miR-1	–	−0.11	0.034
Type II Area/SC (miR-23b)	0.354	0.595	0.003
Fiber type ratio (miR-126)	0.324	0.569	0.002
Fiber type area ratio	0.337	–	0.011
miR-126	–	0.385	0.009
miR-145	–	0.399	0.029

### Discriminant analysis

**Figure 5:**
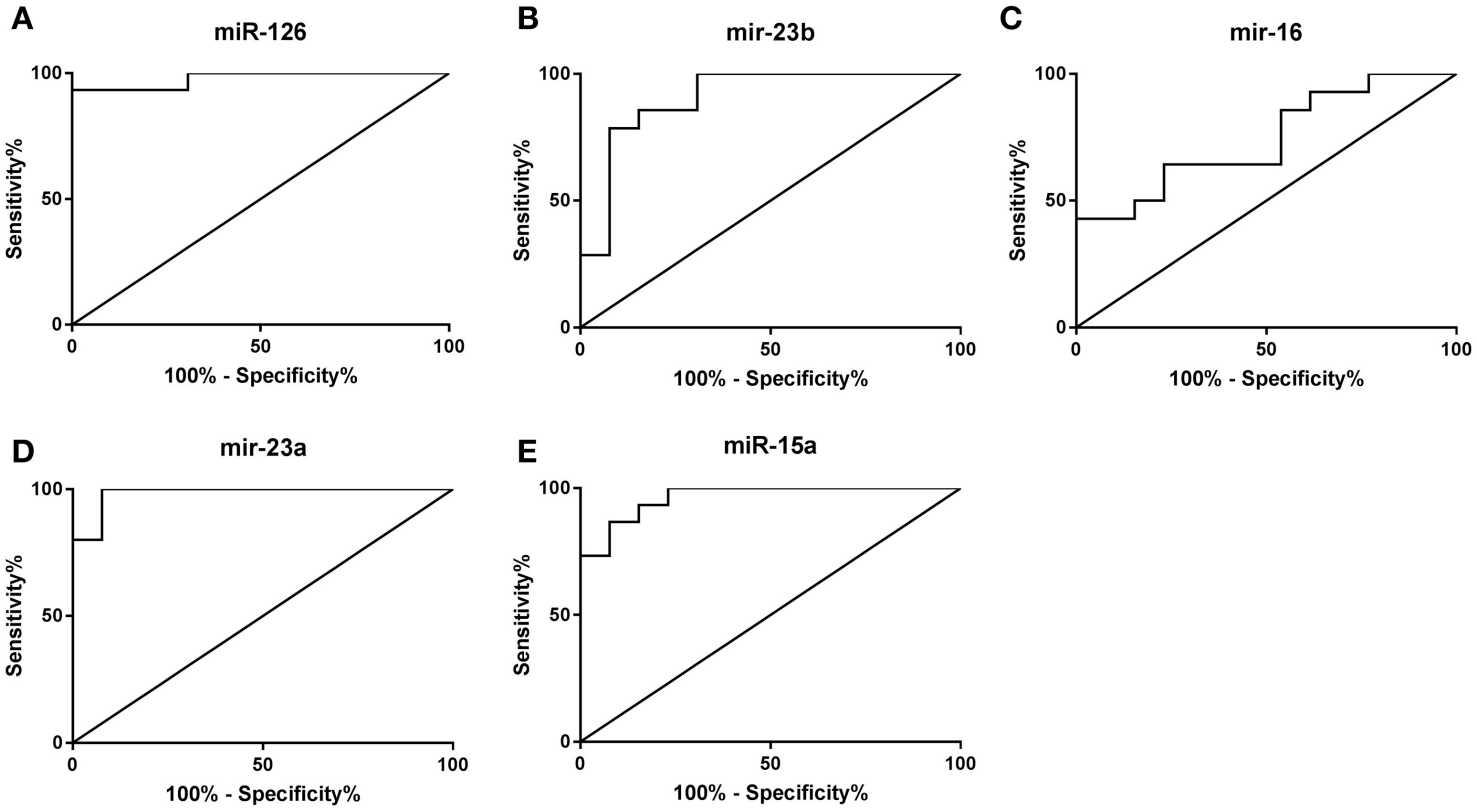
Receiver operator curve analysis to determine sensitivity and specificity. **(A)** miR-126, **(B)** miR-23b, **(C)** miR-16, **(D)** miR-23a, **(E)** miR-15a.

Of the 17 miRs analyzed, stepwise discriminant analyses revealed that the combination of five miRs (miR-126, -23b, -16, -23a, and 15a) correctly classified 100% of participants as powerlifters or controls. Receiver operator characteristic curves were then used to measure the sensitivity and specificity of these miRs (Figure 5). miR-126 differentiated powerlifters with an area under curve of 0.98, sensitivity 93% and specificity 100%. miR-23b distinguished powerlifters with an area under curve of 0.91, sensitivity 85.7% and specificity 84.6% whereas miR-16 had an area under curve of 0.74 with sensitivity 85.7% and specificity 46.2%. miR-23a had an area under curve of 0.98 with sensitivity 100% and specificity 92.3% while miR-15a had an area under the curve of 0.96 with a sensitivity of 93.3% and specificity of 84.6%.

## Discussion

Powerlifters represent an extreme muscular phenotype with muscle fibers double the size of controls and the ability to exert ~45% more torque. These athletes provide a unique opportunity to study how differences in muscle miR and gene expression may regulate the maintenance of the extreme increases in muscle size and strength. A targeted approach was used to analyse miRNA species that have previously been shown to be both differentially regulated within skeletal muscle, corresponding to a change in phenotype, and further have been established to be involved in the mechanistic control of muscle-regulatory genes. Of the 17 miRs analyzed, a total of 12 miRs were differently expressed in the biopsied skeletal muscle samples between powerlifters and healthy controls with miR-126, -23b, -16, -23a and -15a showing the greatest separation between groups. The different miR expression patterns in powerlifters have putative roles in the control of fiber type, protein turnover, muscle remodeling, and angiogenesis.

Chronic resistance training results in increases in fiber area, alteration in muscle architecture and improvements in neural drive which are associated improvements in peak torque (Kawakami et al., 1993; Brechue and Abe, 2002; Matta et al., 2011). The present study demonstrated that both muscle fiber types in powerlifters are approximately two-fold larger than those in healthy controls. This is in contrast to previous analyses indicating a 10 week period of resistance training produces ~12.2% increases in fiber CSA (Häkkinen et al., 1998). The increased myonuclear content in powerlifters were offset by increases in fiber size showing support for myonuclear domain theory in both fiber types (Hawke, 2005; Petrella et al., 2006; Jackson et al., 2012). Whilst area per satellite cell for type I fibers was not different between groups, area per satellite cell in type II fibers was significantly higher (~1.6-fold) in the powerlifters. In studies of untrained individuals who undertook 2–4 months of resistance training resulting in muscle hypertrophy, satellite cells per fiber area were seen to increase (Kadi and Thornell, 2000; Olsen et al., 2006). This finding is inconsistent with the powerlifters in the present study. The greater type II fiber area per satellite cell in the powerlifters may represent an adaptive ceiling not observed in shorter duration training studies.

**Figure 6:**
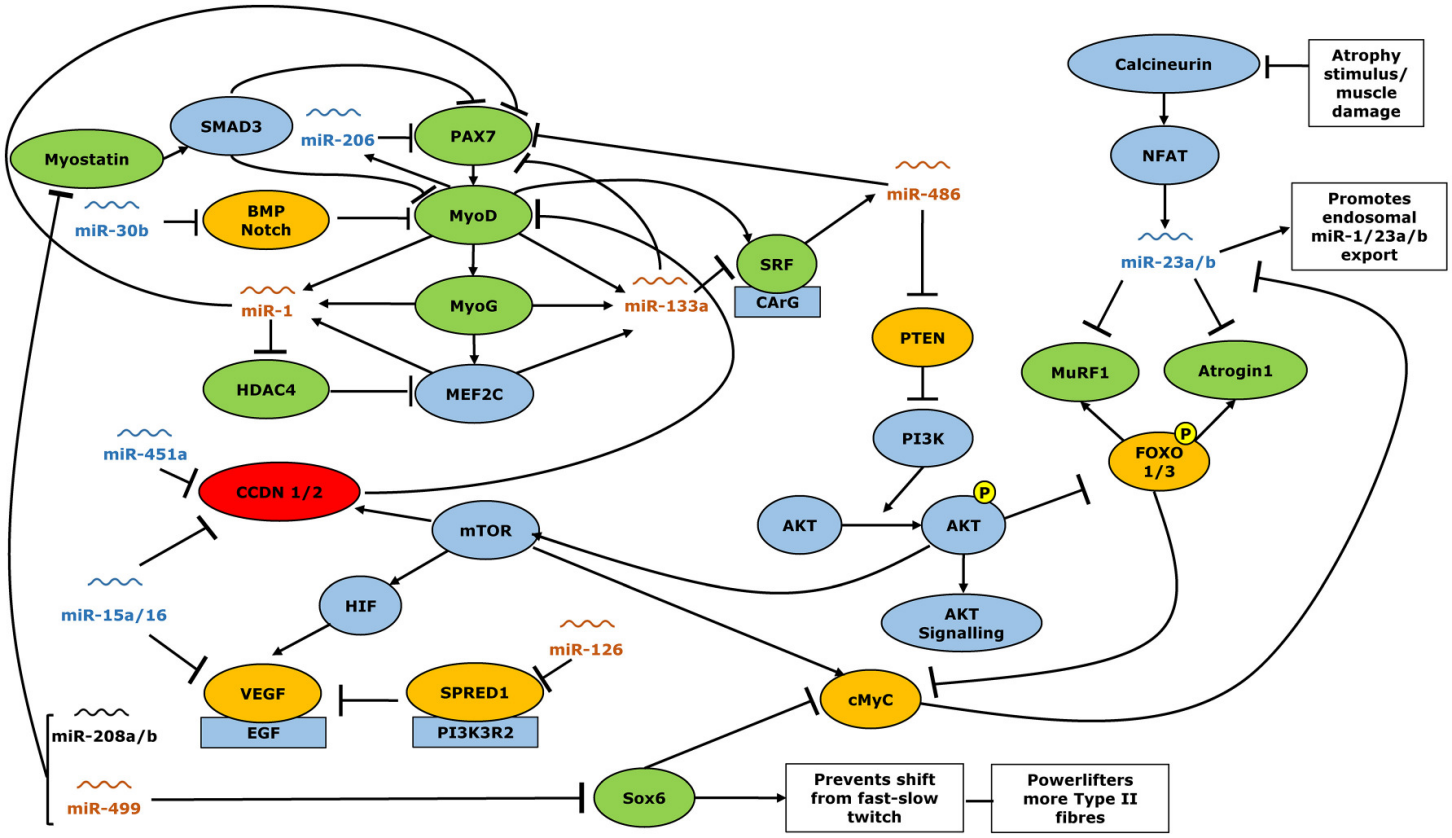
Interactions of miRNA with expression gene expression of muscle function regulators. Orange miRs show greater expression in healthy controls than power lifters, blue miR showed lower expression in controls than power lifters. miRs shown in black were expressed similarly in power lifters and controls. Green genes more abundant in power lifters, red less abundant while yellow were unchanged. Arrowheads indicate a positive interaction while perpendicular lines indicate an inhibitory effect.

The human genome has been shown to encode at least 1,881 non-coding miRNAs (miRbase.org Version 21). Amongst these, the importance of a subset have been identified as playing crucial roles in the myogenesis, hypertrophy, and atrophy of skeletal muscle (Winbanks et al., 2013; Hitachi and Tsuchida, 2014; Soriano-Arroquia et al., 2016). Most of these appear to exert conserved roles across species and have functions from the early phases of myogenesis, from stem cell differentiation through to myofiber atrophy. Within these a subset commonly identified as myomiRs are highly expressed within skeletal muscle (Kovanda et al., 2014) and have reported roles in skeletal muscle maintenance processes (McCarthy, 2014a). We have identified a number of differences in miR expression between powerlifters and controls which through transcriptional regulation (Figure 6) may partially explain the divergent powerlifter phenotype. Several miRNAs were found to correlate with strength, fiber size, type II fiber area per satellite cell and type I:II fiber ratios and area. miR 133a and -486 were significantly correlated against strength and fiber size irrespective of fiber size. miR-206, -1, and 16 were correlated to fiber size alone.

For example, miR-499a has been shown to repress SOX6, a key inhibitor of type II to type I fiber conversion (McCarthy et al., 2009; Quiat et al., 2011; Yeung et al., 2012). Powerlifters showed a greater proportion of type II fibers, reduced miR-499a expression and an increased abundance of SOX6. It is likely that the reduced miR-499a expression allows for a greater SOX6 expression that may support the maintenance of a larger proportion of type II fibers. Since the powerlifter group expressed lower type I/II fiber ratios (0.8 vs. 1.1) they have an abundance of type II fibers compared to controls. This is consistent with other studies that have analyzed the fiber shifts associated with consistent resistance training (Tesch and Karlsson, 1985; Staron et al., 1990) but could also be the result of self-selection to the sport of powerlifting by individuals with a pre-existing abundance of type II fibers. We also find miR-126 that was less abundant in the powerlifters correlated to fiber ratios for cell population and for area representation. Previously it was reported that miR-126 may not be specific to type I fibers (Davidsen et al., 2011). As mentioned earlier since powerlifters have lower type fiber ratios, the idea that miR-126 is type I specific may warrant further analyses. miR-499a is also known to inhibit myostatin, which is a negative regulator of muscle mass (Lee and McPherron, 2001; Whittemore et al., 2003; Drummond et al., 2009). Powerlifters showed more abundant expression of this gene. Myostatin is an upstream regulator of mass accretion that through SMAD3 signaling, can directly inhibit muscle growth and differentiation pathways governed by PAX3/7, MyoD, MyoG, and SRF (Sakuma and Yamaguchi, 2012). The expression of these anabolic factors was significantly higher in powerlifters. Previous training studies indicate a down regulation of myostatin while positive regulators of mass, like MyoD and MyoG, are increased (Kosek et al., 2006; Zanchi et al., 2009; Fernandez-Gonzalo et al., 2013). However, the powerlifters in the present study have undergone years of rigorous training and may not be directly comparable to individuals who have undergone only 2–4 months of resistance training. The expression of other muscle catabolism markers MuRF1 and Atrogin1 showed greater abundance in the powerlifters despite upregulation of their direct inhibitors miR-23a/b. Together these data may be explained by an increased rate of muscle protein turnover in powerlifters compared to controls. It has previously been demonstrated that resistance training increases rates of muscle protein synthesis (Phillips et al., 2002), it is thus conceivable that increase in expression of genes related to both anabolism and catabolism would underpin a greater resting muscle protein turnover in powerlifters as well. It is also possible that the greater expression of catabolic genes such as myostatin, MuRF1 and Atrogin1 in the powerlifters represents a limit to hypertrophic adaptation following long term training which is not seen individuals with lower training ages (Roth et al., 2003).

PAX3/7, MyoD, and MyoG are key components of the myogenesis pathway (Parker et al., 2003; Buckingham and Rigby, 2014) and are more abundantly expressed in powerlifters than the healthy controls. They have been shown to be involved in muscle repair and regeneration in various injury models as well as following resistance exercise (Chakkalakal et al., 2012; Urciuolo et al., 2013). PAX7 protein concentrations are downregulated by miR-1, -133a, -206, and -486 (Chen et al., 2006; Braun and Gautel, 2011). All except miR-486 are transcribed in response to increased MyoD and MyoG expression (Rosenberg et al., 2006; Braun and Gautel, 2011) and act to provide a negative feedback mechanism. miR-1 and -133a were found to be less abundant in powerlifters. The reduced abundance of miR-1 and miR-133a in powerlifters may explain higher HDAC4 and SRF expression, respectively. SRF is expected to promote miR-486 activity via MyoD (Chen et al., 2006; Dey et al., 2011) instead, we observed a lower expression of miR-486 despite elevated SRF expression. miRs -1, -133a, and -486 all directly or indirectly proved negative feedback to the processes of muscle repair and regeneration, despite MyoD and MyoG expression. miRs -1, -133a, and -486 remained down regulated in powerlifters compared to controls. This suggests a complex interaction between multiple miRs and genes related to muscle regeneration.

The cell cycle regulators Cyclin D1 and D2 directly inhibit MyoD and are themselves directly inhibited by miR-451a, -15a, and -16 (Muscat and Dressel, 2000; Aqeilan et al., 2010; Nan et al., 2010). These miRs were more abundant in the powerlifters, perhaps acting to support increased myogenesis and muscle remodeling. CCND2 gene had lower expression in powerlifters likely resulting from the increased expression of its inhibitors miR-451a, -15a, and -16.

Powerlifters exhibited no difference in expression of the proangiogenic gene VEGF which is partly regulated via the mammalian target of rapamycin (mTOR) pathway (Wan et al., 2006). Resistance training induces angiogenesis to support hypertrophy but on its own does not normally increase capillary density (Nederveen et al., 2017). The direct inhibitors of VEGF, miR-15a/16 were however, upregulated in the powerlifters, which may limit angiogenesis in powerlifters. miR-126 which increases VEGF expression via Sprouty-related protein (SPRED1) inhibition (Fish et al., 2008) was lower in powerlifters with no differences in SPRED1 expression between group. The direct regulation of angiogenesis by resistance training via miRs requires further elucidation.

The discriminant analysis identified miR-126, -23b, -16, -23a, and -15a as strong determinants of the powerlifter phenotype, correctly categorizing participants with 100% accuracy. Each of these five miRs independently, correctly group individuals by expression with at least 74% accuracy. The five identified miRs could be investigated further as markers of training adaptation.

The powerlifter phenotype is characterized by dramatic differences in muscle fiber size and force generation capacity when compared to age matched untrained controls. Regulation of mRNA via miR provides a likely mechanism which may underpin these phenotypic differences. However, because protein expression was not measured it is not possible to draw definite conclusions about how miRs might alter phenotype. The design of the study did not allow for the delineation of specific effects of long term resistance training and genetic endowment. Undoubtedly both play a role but it is probable that the majority of the observed effects are due to a long history of high intensity resistance training.

The present study indicates maintenance of the distinct powerlifter phenotype may be modulated by robust differences in miR expression profiles at rest when compared to healthy controls. Specifically, miR-126, -23b, -16, -23a, and -15a discriminate accurately between powerlifters and controls. Differences in miR expression are involved in the regulation of downstream mRNA but can also themselves be regulated by mRNA making understanding the system a complex undertaking. miRs identified from the rare elite powerlifter phenotype can now be investigated in other populations with disparities in muscle strength and size such as young and older adults and those with myopathies in order to validate their importance as biomarkers of muscle function.

## Author contributions

Performed experiments: RD, TB, KA, NZ. Analyzed data: RD, TB. Primer design and pathway illustration: NZ. Drafted manuscript: RD and CM. Critically evaluated and contributed to the manuscript: RD, TB, CM, TR, and DC. CM is responsible for the final content of the manuscript.

### Conflict of interest statement

The authors declare that the research was conducted in the absence of any commercial or financial relationships that could be construed as a potential conflict of interest.

## References

[B1] Ambros V. Bartel B. Bartel D. P. Burge C. B. Carrington J. C. Chen X.. (2003). A uniform system for microRNA annotation. RNA 9, 277–279. 10.1261/rna.2183803 12592000 PMC1370393

[B2] Aqeilan R. Calin G. Croce C. (2010). miR-15a and miR-16-1 in cancer: discovery, function and future perspectives. Cell Death Diff. 17, 215–220. 10.1038/cdd.2009.69 19498445

[B3] Bandi N. Vassella E. (2011). miR-34a and miR-15a/16 are co-regulated in non-small cell lung cancer and control cell cycle progression in a synergistic and Rb-dependent manner. Mol. Cancer 10:55. 10.1186/1476-4598-10-55 21575235 PMC3120797

[B4] Bellamy L. M. Joanisse S. Grubb A. Mitchell C. J. McKay B. R. Phillips S. M.. (2014). The acute satellite cell response and skeletal muscle hypertrophy following resistance training. PLoS ONE 9:e109739. 10.1371/journal.pone.0109739 25313863 PMC4196938

[B5] Braun T. Gautel M. (2011). Transcriptional mechanisms regulating skeletal muscle differentiation, growth and homeostasis. Nat. Rev. Mol. Cell Biol. 12, 349–361. 10.1038/nrm3118 21602905

[B6] Brechue W. F. Abe T. (2002). The role of FFM accumulation and skeletal muscle architecture in powerlifting performance. Eur. J. Appl. Physiol. 86, 327–336. 10.1007/s00421-001-0543-7 11990746

[B7] Buckingham M. Rigby P. W. (2014). Gene regulatory networks and transcriptional mechanisms that control myogenesis. Dev. Cell 28, 225–238. 10.1016/j.devcel.2013.12.020 24525185

[B8] Chakkalakal J. V. Jones K. M. Basson M. A. Brack A. S. (2012). The aged niche disrupts muscle stem cell quiescence. Nature 490, 355–360. 10.1038/nature11438 23023126 PMC3605795

[B9] Chen J.-F. Mandel E. M. Thomson J. M. Wu Q. Callis T. E. Hammond S. M.. (2006). The role of microRNA-1 and microRNA-133 in skeletal muscle proliferation and differentiation. Nat. Genet. 38, 228–233. 10.1038/ng1725 16380711 PMC2538576

[B10] Cordes K. R. Sheehy N. T. White M. P. Berry E. C. Morton S. U. Muth A. N.. (2009). miR-145 and miR-143 regulate smooth muscle cell fate and plasticity. Nature 460, 705–710. 10.1038/nature08195 19578358 PMC2769203

[B11] Davidsen P. K. Gallagher I. J. Hartman J. W. Tarnopolsky M. A. Dela F. Helge J. W.. (2011). High responders to resistance exercise training demonstrate differential regulation of skeletal muscle microRNA expression. J. Appl. Physiol. 110, 309–317. 10.1152/japplphysiol.00901.2010 21030674

[B12] Dey B. K. Gagan J. Dutta A. (2011). miR-206 and-486 induce myoblast differentiation by downregulating Pax7. Mol. Cell. Biol. 31, 203–214. 10.1128/MCB.01009-10 21041476 PMC3019853

[B13] Drummond M. J. Glynn E. L. Fry C. S. Dhanani S. Volpi E. Rasmussen B. B. (2009). Essential amino acids increase microRNA-499,-208b, and-23a and downregulate myostatin and myocyte enhancer factor 2C mRNA expression in human skeletal muscle. J. Nutr. 139, 2279–2284. 10.3945/jn.109.112797 19828686 PMC2777476

[B14] Drummond M. J. McCarthy J. J. Fry C. S. Esser K. A. Rasmussen B. B. (2008). Aging differentially affects human skeletal muscle microRNA expression at rest and after an anabolic stimulus of resistance exercise and essential amino acids. Am. J. Physiol. Endocrinol. Metab. 295, E1333–E1340. 10.1152/ajpendo.90562.2008 18827171 PMC2603551

[B15] Edman K. (1979). The velocity of unloaded shortening and its relation to sarcomere length and isometric force in vertebrate muscle fibres. J. Physiol. 291, 143–159. 10.1113/jphysiol.1979.sp012804 314510 PMC1280892

[B16] Eisenberg E. Levanon E. Y. (2013). Human housekeeping genes, revisited. Trends Genetics 29, 569–574. 10.1016/j.tig.2013.05.010 23810203

[B17] Fernandez-Gonzalo R. Lundberg T. R. Tesch P. A. (2013). Acute molecular responses in untrained and trained muscle subjected to aerobic and resistance exercise training versus resistance training alone. Acta Physiol. 209, 283–294. 10.1111/apha.12174 24112827

[B18] Figueiredo V. C. Roberts L. A. Markworth J. F. Barnett M. P. Coombes J. S. Raastad T.. (2016). Impact of resistance exercise on ribosome biogenesis is acutely regulated by post-exercise recovery strategies. Physiol. Rep. 4:e12670. 10.14814/phy2.12670 26818586 PMC4760384

[B19] Fish J. E. Santoro M. M. Morton S. U. Yu S. Yeh R.-F. Wythe J. D.. (2008). miR-126 regulates angiogenic signaling and vascular integrity. Dev. Cell 15, 272–284. 10.1016/j.devcel.2008.07.008 18694566 PMC2604134

[B20] Gastebois C. Chanon S. Rome S. Durand C. Pelascini E. Jalabert A.. (2016). Transition from physical activity to inactivity increases skeletal muscle miR-148b content and triggers insulin resistance. Physiol. Rep. 4:e12902. 10.14814/phy2.12902 27597765 PMC5027343

[B21] Häkkinen K. Newton R. U. Gordon S. E. McCormick M. Volek J. S. Nindl B. C.. (1998). Changes in muscle morphology, electromyographic activity, and force production characteristics during progressive strength training in young and older men. J. Gerontol. A Biol. Sci. Med. Sci. 53, B415–B423. 10.1093/gerona/53A.6.B415 9823737

[B22] Hawke T. J. (2005). Muscle stem cells and exercise training. Exerc. Sport Sci. Rev. 33, 63–68. 10.1097/00003677-200504000-00002 15821426

[B23] Hitachi K. Tsuchida K. (2014). Role of microRNAs in skeletal muscle hypertrophy. Front. Physiol. 4:408. 10.3389/fphys.2013.00408 24474938 PMC3893574

[B24] Ikeda S. He A. Kong S. W. Lu J. Bejar R. Bodyak N.. (2009). MicroRNA-1 negatively regulates expression of the hypertrophy-associated calmodulin and Mef2a genes. Mol. Cell. Biol. 29, 2193–2204. 10.1128/MCB.01222-08 19188439 PMC2663304

[B25] Jackson J. R. Mula J. Kirby T. J. Fry C. S. Lee J. D. Ubele M. F.. (2012). Satellite cell depletion does not inhibit adult skeletal muscle regrowth following unloading-induced atrophy. Am. J. Physiol. Cell Physiol. 303, C854–C861. 10.1152/ajpcell.00207.2012 22895262 PMC3469717

[B26] Jaric S. (2003). Role of body size in the relation between muscle strength and movement performance. Exerc. Sport Sci. Rev. 31, 8–12. 10.1097/00003677-200301000-00003 12562164

[B27] Kadi F. Thornell L.-E. (2000). Concomitant increases in myonuclear and satellite cell content in female trapezius muscle following strength training. Histochem. Cell Biol. 113, 99–103. 10.1007/s004180050012 10766262

[B28] Kapchinsky S. Mayaki D. Debigare R. Maltais F. Taivassalo T. Hussain S. (2015). Regulation of Muscle Atrophy-related microRNAs in limb muscles of COPD Patients, in B57. Gene Regulation: miRNAs and Epigenetics. (Denver: American Thoracic Society), A3483–A3483.

[B29] Kawakami Y. Abe T. Fukunaga T. (1993). Muscle-fiber pennation angles are greater in hypertrophied than in normal muscles. J. Appl. Physiol. 74, 2740–2744. 8365975 10.1152/jappl.1993.74.6.2740

[B30] Kosek D. J. Kim J.-S. Petrella J. K. Cross J. M. Bamman M. M. (2006). Efficacy of 3 days/wk resistance training on myofiber hypertrophy and myogenic mechanisms in young vs. older adults. J. Appl. Physiol. 101, 531–544. 10.1152/japplphysiol.01474.2005 16614355

[B31] Kovanda A. Režen T. Rogelj B. (2014). MicroRNA in skeletal muscle development, growth, atrophy, and disease. Wiley Interdiscip. Rev. RNA 5, 509–525. 10.1002/wrna.1227 24838768

[B32] Landgraf P. Rusu M. Sheridan R. Sewer A. Iovino N. Aravin A.. (2007). A mammalian microRNA expression atlas based on small RNA library sequencing. Cell 129, 1401–1414. 10.1016/j.cell.2007.04.040 17604727 PMC2681231

[B33] Lee S.-J. McPherron A. C. (2001). Regulation of myostatin activity and muscle growth. Proc. Natl. Acad. Sci. U.S.A. 98, 9306–9311. 10.1073/pnas.151270098 11459935 PMC55416

[B34] Li X. Xi Y. (2011). Predictive capacity and functional significance of MicroRNA in human melanoma, in Research on Melanoma - A Glimpse into Current Directions and Future Trends, ed Murph M. (INTECH Open Access Publisher), 3–18.

[B35] MacDougall J. Sale D. Elder G. C. Sutton J. (1982). Muscle ultrastructural characteristics of elite powerlifters and bodybuilders. Eur. J. Appl. Physiol. Occup. Physiol. 48, 117–126. 10.1007/BF00421171 7199447

[B36] Mackey A. L. Kjaer M. Charifi N. Henriksson J. Bojsen-Moller J. Holm L.. (2009). Assessment of satellite cell number and activity status in human skeletal muscle biopsies. Muscle Nerve 40, 455–465. 10.1002/mus.21369 19705426

[B37] Marcotte G. R. West D. W. Baar K. (2015). The molecular basis for load-induced skeletal muscle hypertrophy. Calcif. Tissue Int. 96, 196–210. 10.1007/s00223-014-9925-9 25359125 PMC4809742

[B38] Margolis L. M. Lessard S. J. Ezzyat Y. Fielding R. A. Rivas D. A. (2016). Circulating MicroRNA are predictive of aging and acute adaptive response to resistance exercise in men. J. Gerontol. A Biol. Sci. Med. Sci. glw243. 10.1093/gerona/glw243 27927764 PMC5861902

[B39] Mathonnet G. Fabian M. R. Svitkin Y. V. Parsyan A. Huck L. Murata T.. (2007). MicroRNA inhibition of translation initiation *in vitro* by targeting the cap-binding complex eIF4F. Science 317, 1764–1767. 10.1126/science.1146067 17656684

[B40] Matta T. Simão R. de Salles B. F. Spineti J. Oliveira L. F. (2011). Strength training's chronic effects on muscle architecture parameters of different arm sites. J. Strength Condit. Res. 25, 1711–1717. 10.1519/JSC.0b013e3181dba162 21602648

[B41] McCarthy J. J. (2011). The MyomiR network in skeletal muscle plasticity. Exerc. Sport Sci. Rev. 39:150. 10.1097/JES.0b013e31821c01e1 21467943 PMC4871711

[B42] McCarthy J. J. (2014a). MicroRNA and skeletal muscle function: novel potential roles in exercise, diseases, and aging. Front. Physiol. 5:290. 10.3389/fphys.2014.00290 25132822 PMC4116776

[B43] McCarthy J. J. (2014b). Out FoxO'd by microRNA. Focus on “miR-182 attenuates atrophy-related gene expression by targeting FoxO3 in skeletal muscle”. Am. Physiol. Soc. 307, C311–C313. 10.1152/ajpcell.00219.2014 24990646

[B44] McCarthy J. J. Esser K. A. (2007). MicroRNA-1 and microRNA-133a expression are decreased during skeletal muscle hypertrophy. J. Appl. Physiol. 102, 306–313. 10.1152/japplphysiol.00932.2006 17008435

[B45] McCarthy J. J. Esser K. A. Peterson C. A. Dupont-Versteegden E. E. (2009). Evidence of MyomiR network regulation of β-myosin heavy chain gene expression during skeletal muscle atrophy. Physiol. Genomics 39, 219–226. 10.1152/physiolgenomics.00042.2009 19690046 PMC2789671

[B46] McCarthy J. J. Mula J. Miyazaki M. Erfani R. Garrison K. Farooqui A. B.. (2011). Effective fiber hypertrophy in satellite cell-depleted skeletal muscle. Development 138, 3657–3666. 10.1242/dev.068858 21828094 PMC3152923

[B47] Mercken E. M. Majounie E. Ding J. Guo R. Kim J. Bernier M.. (2013). Age-associated miRNA alterations in skeletal muscle from rhesus monkeys reversed by caloric restriction. Aging (Albany, NY). 5, 692–703. 10.18632/aging.100598 24036467 PMC3808701

[B48] Muscat G. E. Dressel U. (2000). Not a minute to waste. Nat. Med. 6, 1216–1217. 10.1038/81312 11062529

[B49] Musumeci M. Coppola V. Addario A. Patrizii M. Maugeri-Sacca M. Memeo L.. (2011). Control of tumor and microenvironment cross-talk by miR-15a and miR-16 in prostate cancer. Oncogene 30, 4231–4242. 10.1038/onc.2011.140 21532615

[B50] Naguibneva I. Ameyar-Zazoua M. Polesskaya A. Ait-Si-Ali S. Groisman R. Souidi M.. (2006). The microRNA miR-181 targets the homeobox protein Hox-A11 during mammalian myoblast differentiation. Nat. Cell Biol. 8, 278–284. 10.1038/ncb1373 16489342

[B51] Nakasa T. Ishikawa M. Shi M. Shibuya H. Adachi N. Ochi M. (2010). Acceleration of muscle regeneration by local injection of muscle-specific microRNAs in rat skeletal muscle injury model. J. Cell. Mol. Med. 14, 2495–2505. 10.1111/j.1582-4934.2009.00898.x 19754672 PMC3823166

[B52] Nan Y. Han L. Zhang A. Wang G. Jia Z. Yang Y.. (2010). MiRNA-451 plays a role as tumor suppressor in human glioma cells. Brain Res. 1359, 14–21. 10.1016/j.brainres.2010.08.074 20816946

[B53] Narici M. V. Roi G. Landoni L. Minetti A. Cerretelli P. (1989). Changes in force, cross-sectional area and neural activation during strength training and detraining of the human quadriceps. Eur. J. Appl. Physiol. Occup. Physiol. 59, 310–319. 10.1007/BF02388334 2583179

[B54] Nederveen J. P. Snijders T. Joanisse S. Wavell C. G. Mitchell C. J. Johnston L. M.. (2017). Altered muscle satellite cell activation following 16 wk of resistance training in young men. Am. J. Physiol. Regul. Integr. Comp. Physiol. 312, R85–R92. 10.1152/ajpregu.00221.2016 27834290 PMC5283938

[B55] Nielsen J. L. Aagaard P. Bech R. D. Nygaard T. Hvid L. G. Wernbom M.. (2012). Proliferation of myogenic stem cells in human skeletal muscle in response to low-load resistance training with blood flow restriction. J. Physiol. 590, 4351–4361. 10.1113/jphysiol.2012.237008 22802591 PMC3473290

[B56] Nielsen M. Hansen J. Hedegaard J. Nielsen R. Panitz F. Bendixen C.. (2010). MicroRNA identity and abundance in porcine skeletal muscles determined by deep sequencing. Anim. Genet. 41, 159–168. 10.1111/j.1365-2052.2009.01981.x 19917043

[B57] Olsen S. Aagaard P. Kadi F. Tufekovic G. Verney J. Olesen J. L.. (2006). Creatine supplementation augments the increase in satellite cell and myonuclei number in human skeletal muscle induced by strength training. J. Physiol. 573, 525–534. 10.1113/jphysiol.2006.107359 16581862 PMC1779717

[B58] Ostrander E. A. Huson H. J. Ostrander G. K. (2009). Genetics of athletic performance. Annu. Rev. Genomics Hum. Genet. 10, 407–429. 10.1146/annurev-genom-082908-150058 19630564

[B59] Parker M. H. Seale P. Rudnicki M. A. (2003). Looking back to the embryo: defining transcriptional networks in adult myogenesis. Nat. Rev. Genet. 4, 497–507. 10.1038/nrg1109 12838342

[B60] Petrella J. K. Kim J.-S. Cross J. M. Kosek D. J. Bamman M. M. (2006). Efficacy of myonuclear addition may explain differential myofiber growth among resistance-trained young and older men and women. Am. J. Physiol. Endocrinol. Metab. 291, E937–E946. 10.1152/ajpendo.00190.2006 16772322

[B61] Petrella J. K. Kim J.-S. Mayhew D. L. Cross J. M. Bamman M. M. (2008). Potent myofiber hypertrophy during resistance training in humans is associated with satellite cell-mediated myonuclear addition: a cluster analysis. J. Appl. Physiol. 104, 1736–1742. 10.1152/japplphysiol.01215.2007 18436694

[B62] Phillips S. M. Parise G. Roy B. D. Tipton K. D. Wolfe R. R. Tamopolsky M. A. (2002). Resistance-training-induced adaptations in skeletal muscle protein turnover in the fed state. Can. J. Physiol. Pharmacol. 80, 1045–1053. 10.1139/y02-134 12489923

[B63] Pitsiladis Y. Wang G. Wolfarth B. Scott R. Fuku N. Mikami E.. (2013). Genomics of elite sporting performance: what little we know and necessary advances. Br. J. Sports Med. 47, 550–555. 10.1136/bjsports-2013-092400 23632745

[B64] Quiat D. Voelker K. A. Pei J. Grishin N. V. Grange R. W. Bassel-Duby R.. (2011). Concerted regulation of myofiber-specific gene expression and muscle performance by the transcriptional repressor Sox6. Proc. Natl. Acad. Sci. U.S.A. 108, 10196–10201. 10.1073/pnas.1107413108 21633012 PMC3121857

[B65] Režen T. Kovanda A. Eiken O. Mekjavic I. Rogelj B. (2014). Expression changes in human skeletal muscle miRNAs following 10 days of bed rest in young healthy males. Acta Physiol. 210, 655–666. 10.1111/apha.12228 24410893

[B66] Roberts L. A. Raastad T. Markworth J. F. Figueiredo V. C. Egner I. M. Shield A.. (2015). Post-exercise cold water immersion attenuates acute anabolic signalling and long-term adaptations in muscle to strength training. J. Physiol. 593, 4285–4301. 10.1113/JP270570 26174323 PMC4594298

[B67] Rosenberg M. I. Georges S. A. Asawachaicharn A. Analau E. Tapscott S. J. (2006). MyoD inhibits Fstl1 and Utrn expression by inducing transcription of miR-206. J. Cell Biol. 175, 77–85. 10.1083/jcb.200603039 17030984 PMC2064500

[B68] Roth S. M. Martel G. F. Ferrell R. E. Metter E. J. Hurley B. F. Rogers M. A. (2003). Myostatin gene expression is reduced in humans with heavy-resistance strength training: a brief communication. Exp. Biol. Med. 228, 706–709. 10.1177/153537020322800609 12773702

[B69] Sachdeva M. Zhu S. Wu F. Wu H. Walia V. Kumar S.. (2009). p53 represses c-Myc through induction of the tumor suppressor miR-145. Proc. Natl. Acad. Sci. U.S.A. 106, 3207–3212. 10.1073/pnas.0808042106 19202062 PMC2651330

[B70] Sakuma K. Yamaguchi A. (2012). Molecular and cellular mechanism of muscle regeneration, in Skeletal Muscle - From Myogenesis to Clinical Relations, ed Cseri J. (INTECH Open Access Publisher), 3–30.

[B71] Schmittgen T. D. Livak K. J. (2008). Analyzing real-time PCR data by the comparative CT method. Nat. Protoc. 3, 1101–1108. 10.1038/nprot.2008.73 18546601

[B72] Small E. M. O'Rourke J. R. Moresi V. Sutherland L. B. McAnally J. Gerard R. D.. (2010). Regulation of PI3-kinase/Akt signaling by muscle-enriched microRNA-486. Proc. Natl. Acad. Sci. U.S.A. 107, 4218–4223. 10.1073/pnas.1000300107 20142475 PMC2840099

[B73] Soriano-Arroquia A. House L. Tregilgas L. Canty-Laird E. Goljanek-Whysall K. (2016). The functional consequences of age-related changes in microRNA expression in skeletal muscle. Biogerontology 17, 641–654. 10.1007/s10522-016-9638-8 26922183 PMC4889642

[B74] Staron R. Malicky E. Leonardi M. Falkel J. Hagerman F. Dudley G. (1990). Muscle hypertrophy and fast fiber type conversions in heavy resistance-trained women. Eur. J. Appl. Physiol. Occup. Physiol. 60, 71–79. 10.1007/BF00572189 2311599

[B75] Sun C.-Y. She X.-M. Qin Y. Chu Z.-B. Chen L. Ai L.-S.. (2012). miR-15a and miR-16 affect the angiogenesis of multiple myeloma by targeting VEGF. Carcinogenesi 34, 426–435. 10.1093/carcin/bgs333 23104180

[B76] Tesch P. Karlsson J. (1985). Muscle fiber types and size in trained and untrained muscles of elite athletes. J. Appl. Physiol. 59, 1716–1720. 4077779 10.1152/jappl.1985.59.6.1716

[B77] Tonevitsky A. G. Maltseva D. V. Abbasi A. Samatov T. R. Sakharov D. A. Shkurnikov M. U.. (2013). Dynamically regulated miRNA-mRNA networks revealed by exercise. BMC Physiol. 13:9. 10.1186/1472-6793-13-9 24219008 PMC3681679

[B78] Townley-Tilson W. D. Callis T. E. Wang D. (2010). MicroRNAs 1, 133, and 206: critical factors of skeletal and cardiac muscle development, function, and disease. Int. J. Biochem. Cell Biol. 42, 1252–1255. 10.1016/j.biocel.2009.03.002 20619221 PMC2904322

[B79] Urciuolo A. Quarta M. Morbidoni V. Gattazzo F. Molon S. Grumati P.. (2013). Collagen VI regulates satellite cell self-renewal and muscle regeneration. Nat. Commun. 4, 1964. 10.1038/ncomms2964 23743995 PMC3682802

[B80] Vandesompele J. De Preter K. Pattyn F. Poppe B. Van Roy N. De Paepe A.. (2002). Accurate normalization of real-time quantitative RT-PCR data by geometric averaging of multiple internal control genes. Genome Biol. 3:research0034. 10.1186/gb-2002-3-7-research0034 12184808 PMC126239

[B81] van Rooij E. Quiat D. Johnson B. A. Sutherland L. B. Qi X. Richardson J. A.. (2009). A family of microRNAs encoded by myosin genes governs myosin expression and muscle performance. Dev. Cell 17, 662–673. 10.1016/j.devcel.2009.10.013 19922871 PMC2796371

[B82] Wada S. Kato Y. Okutsu M. Miyaki S. Suzuki K. Yan Z.. (2011). Translational suppression of atrophic regulators by microRNA-23a integrates resistance to skeletal muscle atrophy. J. Biol. Chem. 286, 38456–38465. 10.1074/jbc.M111.271270 21926429 PMC3207415

[B83] Walden T. B. Timmons J. A. Keller P. Nedergaard J. Cannon B. (2009). Distinct expression of muscle-specific MicroRNAs (myomirs) in brown adipocytes. J. Cell. Physiol. 218, 444–449. 10.1002/jcp.21621 18937285

[B84] Wan X. Shen N. Mendoza A. Khanna C. Helman L. J. (2006). CCI-779 inhibits rhabdomyosarcoma xenograft growth by an antiangiogenic mechanism linked to the targeting of mTOR/Hif-1α/VEGF signaling. Neoplasia 8, 394–401. 10.1593/neo.05820 16790088 PMC1592447

[B85] Wang S. Aurora A. B. Johnson B. A. Qi X. McAnally J. Hill J. A.. (2008). The endothelial-specific microRNA miR-126 governs vascular integrity and angiogenesis. Dev. Cell 15, 261–271. 10.1016/j.devcel.2008.07.002 18694565 PMC2685763

[B86] Wang X. H. (2013). MicroRNA in myogenesis and muscle atrophy. Curr. Opin. Clin. Nutr. Metab. Care 16:258. 10.1097/MCO.0b013e32835f81b9 23449000 PMC3967234

[B87] Whittemore L.-A. Song K. Li X. Aghajanian J. Davies M. Girgenrath S.. (2003). Inhibition of myostatin in adult mice increases skeletal muscle mass and strength. Biochem. Biophys. Res. Commun. 300, 965–971. 10.1016/S0006-291X(02)02953-4 12559968

[B88] Williams A. H. Valdez G. Moresi V. Qi X. McAnally J. Elliott J. L.. (2009). MicroRNA-206 delays ALS progression and promotes regeneration of neuromuscular synapses in mice. Science 326, 1549–1554. 10.1126/science.1181046 20007902 PMC2796560

[B89] Winbanks C. E. Beyer C. Hagg A. Qian H. Sepulveda P. V. Gregorevic P. (2013). miR-206 represses hypertrophy of myogenic cells but not muscle fibers via inhibition of HDAC4. PLoS ONE 8:e73589. 10.1371/journal.pone.0073589 24023888 PMC3759420

[B90] Yablonka-Reuveni Z. Rudnicki M. A. Rivera A. J. Primig M. Anderson J. E. Natanson P. (1999). The transition from proliferation to differentiation is delayed in satellite cells from mice lacking MyoD. Dev. Biol. 210, 440–455. 10.1006/dbio.1999.9284 10357902 PMC5027208

[B91] Yeung F. Chung E. Guess M. G. Bell M. L. Leinwand L. A. (2012). Myh7b/miR-499 gene expression is transcriptionally regulated by MRFs and Eos. Nucleic Acids Res. 40, 7303–7318. 10.1093/nar/gks466 22638570 PMC3424578

[B92] Yin H. Price F. Rudnicki M. A. (2013). Satellite cells and the muscle stem cell niche. Physiol. Rev. 93, 23–67. 10.1152/physrev.00043.2011 23303905 PMC4073943

[B93] Yin K.-J. Olsen K. Hamblin M. Zhang J. Schwendeman S. P. Chen Y. E. (2012). Vascular endothelial cell-specific microRNA-15a inhibits angiogenesis in hindlimb ischemia. J. Biol. Chem. 287, 27055–27064. 10.1074/jbc.M112.364414 22692216 PMC3411046

[B94] Zacharewicz E. Lamon S. Russell A. P. (2013). MicroRNAs in skeletal muscle and their regulation with exercise, ageing, and disease. Front. Physiol. 4:266. 10.3389/fphys.2013.00266 24137130 PMC3786223

[B95] Zanchi N. E. de Siqueira Filho M. A. Lira F. S. Rosa J. C. Yamashita A. S. de Oliveira Carvalho C. R.. (2009). Chronic resistance training decreases MuRF-1 and Atrogin-1 gene expression but does not modify Akt, GSK-3β and p70S6K levels in rats. Eur. J. Appl. Physiol. 106, 415–423. 10.1007/s00421-009-1033-6 19306017

[B96] Zang W.-Q. Yang X. Wang T. Wang Y.-Y. Du Y.-W. Chen X.-N.. (2015). MiR-451 inhibits proliferation of esophageal carcinoma cell line EC9706 by targeting CDKN2D and MAP3K1. World J. Gastroenterol. 21, 5867–5876. 10.3748/wjg.v21.i19.5867 26019450 PMC4438020

