# Supplementary material for: MicroRNAs in Muscle: Characterizing the Powerlifter Phenotype
Source: Front Physiol. 2017 Jun 7;8:383. doi: 10.3389/fphys.2017.00383 (PMC5461344; doi:10.3389/fphys.2017.00383)
Supplement: Supplementary file 1 [file Table1.DOCX]

| miR | ID Number | |
| --- | --- | --- |
| miR-15a-5p | 477858_mir |  |
| miR-16-5p | 477860_mir |  |
| miR-23a-3p | 478532_mir |  |
| miR-23b-3p | 478602_mir |  |
| miR-451a | 477968_mir |  |
| miR-486-5p | 478128_mir |  |
| miR-126-3p | 477887_mir |  |
| miR-133a-3p | 478511_mir |  |
| miR-206 | 477968_mir |  |
| miR-1-3p | 477820_mir |  |
| miR-148b-3p | 477806_mir |  |
| miR-30b-5p | 478007_mir |  |
| miR-145-5p | 477916_mir |  |
| miR-499a-3p | 478948_mir |  |
| miR-208a-3p | 477819_mir |  |
| miR-208b-3p | 477806_mir |  |
| miR-186-5p | 477940_mir |  |
| miR-320a | 478594_mir |  |
| miR-361-5p | 478056_mir |  |

**Supplementary Table 1.** Catalogue numbers for the miRNAs analysed and housekeepers with Thermo Fisher Scientific independent miR assay IDs
