# Supplementary material for: MicroRNAs in Muscle: Characterizing the Powerlifter Phenotype
Source: Front Physiol. 2017 Jun 7;8:383. doi: 10.3389/fphys.2017.00383 (PMC5461344; doi:10.3389/fphys.2017.00383)
Supplement: Supplementary file 2 [file Table2.DOCX]

| **Gene** | **Sequence** |
| --- | --- |
| c-MYC Forward | GGTAGTGGAAAACCAGCAGCC |
| c-MYC Reverse | TCTCCTCCTCGTCGCAGTA |
| MYOD (Forward) | CGGCATGATGGACTACAGCG |
| MYOD (Reverse) | CAGGCAGTCTAGGCTCGAC |
| FOXO 3 (Forward) | TGAACGTGGGGAACTTCACT |
| FOXO 3 (Reverse) | GTGTCAGTTTGAGGGTCTGC |
| PAX 7 (Forward) | CCTTTGGAAGTGTCCACCCC |
| PAX 7 (Reverse) | TCGCCCATTGATGAAGACCC |
| CCND1 (Forward) | GCTGCGAAGTGGAAACCATC |
| CCND1 (Reverse) | CCTCCTTCTGCACACATTTGAA |
| CCND2 (Forward) | CTGCCCCCACCTAGATCATA |
| CCND2 (Reverse) | TCCCTTATGCTGTACTTCAAATAGG |
| MYOG (Forward) | GGCCAAACTTTTGCAGTGAATATT |
| MYOG (Reverse) | TCGGATGGCAGCTTTACAAACAAC |
| NCAM 1 (Forward) | GCAGCGAAGAAAAGACTCTGG |
| NCAM 1 (Reverse) | GCAGATGTACTCTCCGGCAT |
| ATROGIN1 (Forward) | GCAGCTGAACAACATTCAGATCAC |
| ATROGIN1 (Reverse) | CAGCCTCTGCATGATGTTCAGT |
| MuRF1 (Forward) | CCTGAGAGCCATTGACTTTGG |
| MuRF1 (Reverse) | CTTCCCTTCTGTGGACTCTTCCT |
| VEGF (Forward) | TCTTCAAGCCATCCTGTGT |
| VEGF (Reverse) | CTTTCTTTGGTCTGCATTC |
| Myostatin (Forward) | CTACAACGGAAACAATCATTACCA |
| Myostatin (Reverse) | GTTTCAGAGATCGGATTCCAGTAT |
| HDAC4 (Forward) | GGGAGCTGAAGAATGGCTTTG |
| HDAC4 (Reverse) | AGGATCTTGCTCACGCTCAA |
| BMP2 (Forward) | AACGGACATTCGGTCCTTGC |
| BMP2 (Reverse) | CCATGGTCGACCTTTAGGAGA |
| Sox6 (Forward) | GCAAGAACAGATTGCGAGAC |
| Sox6 (Reverse) | AATTGGGATCATGAGCGGAGG |
| PTEN (Forward) | TGTAAAGCTGGAAAGGGACGA |
| PTEN (Reverse) | GGGAATAGTTACTCCCTTTTTGTC |
| SRF (Forward) | CTCAACTCGCCAGACTCTCC |
| SRF (Reverse) | AGTGTGTCCTTGGTCTCCCC |
| SPRED1 (Forward) | CGTTTCAAAGTCCTGCTGATG |
| SPRED1 (Reverse) | CATTTGCTTGTAAGTCATCTGCCC |
| PAX3 (Forward) | CTCACCTCAGGTAATGGGACT |
| PAX3 (Reverse) | GAGCGCGTAATCAGTCTGGG |
| FOXO 1 (Forward) | ACGAGTGGATGGTCAAGAGC |
| FOXO 1 (Reverse) | AATTGAATTCTTCCAGCCCGC |

**Supplementary Table 2.** Forward and reverse mRNAs sequences of analysed genes
