# Supplementary material for: MicroRNAs in Muscle: Characterizing the Powerlifter Phenotype
Source: Front Physiol. 2017 Jun 7;8:383. doi: 10.3389/fphys.2017.00383 (PMC5461344; doi:10.3389/fphys.2017.00383)
Supplement: Supplementary file 3 [file Table3.DOCX]

| **Gene** | **Sequence** |
| --- | --- |
| *EMC7 (Forward)* | GGGCTGGACAGACTTTCTAATG |
| *EMC7 (Reverse)* | CTCCATTTCCCGTCTCATGTCAG |
| *VCP (Forward)* | AAACTCATGGCGAGGTGGAG |
| *VCP (Reverse)* | TGTCAAAGCGACCAAATCGC |
| *CHMP2A (Forward)* | CGCTATGTGCGCAAGTTTGT |
| *CHMP2A (Reverse)* | GGGGCAACTTCAGCTGTCTG |
| *C1orf43 (Forward)* | CTATGGGACAGGGGTCTTTGG |
| *C1orf43 (Reverse)* | TTTGGCTGCTGACTGGTGAT |

**Supplementary Table 3.** Forward and reverse sequences of reference mRNAs
