# Supplementary material for: MicroRNAs in Muscle: Characterizing the Powerlifter Phenotype
Source: Front Physiol. 2017 Jun 7;8:383. doi: 10.3389/fphys.2017.00383 (PMC5461344; doi:10.3389/fphys.2017.00383)
Supplement: Supplementary file 4 [file Table4.DOCX]

| Gene/miR | P-Value | Q*-Value | P<Q* |
| --- | --- | --- | --- |
| miR-126 | 1.194E-06 | 0.0013514 | TRUE |
| miR-133a | 0.0001012 | 0.0027027 | TRUE |
| Myostatin | 0.0001073 | 0.0040541 | TRUE |
| miR-23a | 0.0001246 | 0.0054054 | TRUE |
| miR-23b | 0.0001533 | 0.0067568 | TRUE |
| SRF | 0.0003873 | 0.0081081 | TRUE |
| Pax7 | 0.0004376 | 0.0094595 | TRUE |
| ZFAS1 | 0.0004376 | 0.0108108 | TRUE |
| miR-15a | 0.000489 | 0.0121622 | TRUE |
| HDAC4 | 0.0014381 | 0.0135135 | TRUE |
| MyoG | 0.0016226 | 0.0148649 | TRUE |
| miR-486 | 0.002736 | 0.0162162 | TRUE |
| NCAM | 0.0041465 | 0.0175676 | TRUE |
| Atrogin1 | 0.0043034 | 0.0189189 | TRUE |
| PAX3 | 0.0076647 | 0.0202703 | TRUE |
| miR-1-3p | 0.0083747 | 0.0216216 | TRUE |
| SOX6 | 0.0084403 | 0.022973 | TRUE |
| miR-206 | 0.0094524 | 0.0243243 | TRUE |
| myoD | 0.0107511 | 0.0256757 | TRUE |
| miR-499a | 0.0115594 | 0.027027 | TRUE |
| miR-30b | 0.0127352 | 0.0283784 | TRUE |
| MuRF1 | 0.0129013 | 0.0297297 | TRUE |
| miR-16 | 0.015872 | 0.0310811 | TRUE |
| miR-451a | 0.0173291 | 0.0324324 | TRUE |
| CCND2 | 0.018976 | 0.0337838 | TRUE |
| Vegf | 0.0641044 | 0.0351351 | FALSE |
| miR-208a | 0.0713695 | 0.0364865 | FALSE |
| SPRED1 | 0.0834647 | 0.0378378 | FALSE |
| CCND1 | 0.1978214 | 0.0391892 | FALSE |
| FOXO3a | 0.2134608 | 0.0405405 | FALSE |
| FOXO1 | 0.2441532 | 0.0418919 | FALSE |
| miR-148b | 0.2808157 | 0.0432432 | FALSE |
| PTEN | 0.3016128 | 0.0445946 | FALSE |
| miR-208b | 0.4956834 | 0.0459459 | FALSE |
| c-MYC | 0.6931612 | 0.0472973 | FALSE |
| BMP2 | 0.71763 | 0.0486486 | FALSE |
| miR-145 | 0.7296115 | 0.05 | FALSE |

**Supplementary Table 4.** False discovery rate for multiple gene comparisons
